# Editorial: The challenge of palliative psychology across the lifespan: Between new health emergencies and paradigm shifts

**DOI:** 10.3389/fpsyg.2022.1028330

**Published:** 2022-10-17

**Authors:** Ines Testoni, Hod Orkibi, Franca Benini, Efrat Dagan

**Affiliations:** ^1^FISPPA Department, University of Padova, Padua, Italy; ^2^Drama and Health Science Lab, School of Creative Arts Therapies, Faculty of Social Welfare and Health Sciences, University of Haifa, Haifa, Israel; ^3^Cheryl Spencer Department of Nursing, Faculty of Social Welfare and Health Sciences, University of Haifa, Haifa, Israel; ^4^Department of Paediatrics, Paediatric Pain and Palliative Care Service, University of Padua, Padua, Italy

**Keywords:** palliative psychology, palliative care, loss, grief, model of intervention

Palliative care is an interdisciplinary care service which has been rapidly expanding in health care settings, differentiated between two levels. The first level is administered in hospitals and network care facilities. It concerns interventions to reduce psychophysical pain and distress caused by serious illness, and/or trauma, and the management of suffering caused by disabilities, chronic disease, or other threatening conditions. The second level involves more specifically palliative intervention in end-of-life, hospice and network health care.

Palliative psychology is in a prime position to help patients, their families, social services, and other health professionals coordinate care along the disease continuum to maximize quality of life in critical and end of life situations (Strada, 2017; Testoni et al., 2020). Indeed, palliative health professionals can offer specific competencies, clinical diagnoses of psychological difficulties and perform patient assessments. Palliative health professionals can also deliver empirically-informed counseling, psychotherapy, and assist patients with treatment planning. Further, they deliver staff support services and conduct research on program development and treatment effectiveness. Collaborating with social services, they support patients and families in the managing related difficulties.

However, several barriers exist intervention into palliative care such as the lack of inclusion of these professionals on palliative care teams, gaps in palliative care training, lack of research in the field. Therefore, this Special Topic of Frontiers in Psychology aimed to describe on the one hand the benefits of psychological work in palliative care contexts and welcome empirical findings regarding what psychology can offer in this expanding field. On the other hand, it offers new model and instruments for psychological palliative intervention. All the articles guarantee high-quality theoretical contribution in this field.

The volume is divided into three main sections. The first area is composed by five articles that reflect on death, grief and how to manage the dying and bereavement processes. The first contribution is by Laranjeira et al., titled “*Death Café as a strategy to foster compassionate communities: contributions for death and grief literacy*.” The authors describe the death-positive movement and the most recent manifestation of the death awareness movement, aimed at removing the “death taboo” that characterized the contemporary society. The second article of this first area, by Testoni et al., is inherent to bereavement. The article “*The impact of the COVID-19 pandemic on perinatal loss among Italian couples: A mixed-method study*,” describes the dramatic experience of perinatal loss during the COVID-19 pandemic, utilizing a mixed method research design. Results show that participants suffered from important distress symptoms and needed a psychological support. Moriconi and Cantero-García wrote the third article, titled “*Bereavement groups: understanding grief in parents of children with cancer*” considers the dramatic experience of a child's death, which is the most stressful event and the most complex grief that families face. The main aim is to highlight the needs of bereaved parents, in order to increase the specificity and effectiveness of the therapeutic approach to prevent complications in the process of loss-making. In this same line, the fourth study titled “*Explore the bereavement needs of families of children with cancer from the perspective of caregivers: A qualitative study*,” by Pakseresht et al., considers psychological, social and spiritual reactions in the parents of children with cancer. Results show that the families of these children need to receive a particular bereavement service. It recommends that members of the health care team be trained in assessing family needs, identifying risks of adverse outcomes, continuing care, and providing resources during bereavement. The fifth study titled “*Caregivers' grief in acquired non-death interpersonal loss (NoDIL): A process based model with implications for theory, research and intervention*” by Yehene et al. proposes a process-based model which addresses cognitive-emotional-behavioral challenges caregivers meet in the face of their difficult reality. These require a revision of the interpersonal schemas and the relationships that takes into account the ongoing interactions with the affected family member. The six contribution is focused on the place of death. In the article “*Levels and determinants of place of-death congruence in palliative patients: a systematic review*” by García-Sanjuán et al. reflects on the agreement between the patient's preferred place of death and their actual place of death. Their systematic literature review shows that treatment-related factors such as physical pain control, marital status, having a non-working relative, age, discussing preferred place of death with a healthcare professional, and caregiver's preference have been associated with higher levels of congruence. In the same thematic line, the article “*Preferred place of death in cancer patients: A systematic review and meta-analysis*” by Fereidouni, Rassouli et al. is specifically focused on the preferred place of death and the factors affecting it for adult patients with cancer. The systematic review and meta-analysis showed that more than half of cancer patients chose home as their preferred place of death. Therefore, guided policies need to ensure that the death of the patients in the preferred place should be considered with priority.

The second area, concerning new instruments for the assessment of psychological palliative care, is composed by five articles. The first one, titled “*Screening for distress in oncological patients: The revised version of the psychological distress inventory (PDI-R)*” by Rossi et al., presents the evaluation of the psychometric properties of the PDI and providing a revised version of the tool, offering a solid factorial structure. Results show that the PDI-R is a reliable measure of psychological distress in different samples of oncological patients, with stronger psychometric properties than the original version. Nir et al., present the second article, titled “*Psychometric properties of the Persian version of palliative outcome scale (POS)*” that examined the psychometric properties of translated version of this instrument, showing that the Persian version is a valid and reliable tool and can be used by the clinician to monitor the consequences of palliative care in Iranian cancer patients. The third article by Fereidouni, Ebadi et al., titled “*Psychometric properties of the Persian version of quality of life during serious illness-family caregiver Version 3 (QOLLTI-F V3) in COVID-19 Patients*” is aimed to indicate the psychometric properties of the Persian version of the Quality of Life in Life-Threatening Illness–Family Carer Version (QOLLTI-F) in patients with COVID-19. Results confirm that this instrument may be utilized in clinical trials and research to enhance the quality of life for family care in Iranian society. The fourth article “*Loneliness in bereavement: Measurement matters*” by Vedder et al., reviews the scientific literature to examine how loneliness after bereavement has been operationalized and measured. Results show major disparities and conclusion is that, in selecting a loneliness measure, health care professionals should come to their own well-informed decision, aided by the information provided in the review. In the fifth article “*The personal wellbeing index in Spanish palliative care professional: A cross-sectional study of wellbeing*,” by Pérez-Belmonte et al., aimed to study the Personal Wellbeing Index (PWI) in a sample of Spanish palliative care professionals, as well as to study their levels of well-being and the relationships of well-being with variables such as gender, age, marital status, profession, and professional quality of life, showing that the PWI is adequate to measure personal well-being in Spanish palliative care professionals.

The third section indicates some innovation in the area of interventions. The first article, titled “*How do we talk with people living with dementia about future care: a scoping review*” by Visser et al., reviewed the existing research on practical communication aspects related to dementia in ACP conversations, in order to provide practical suggestions for healthcare professionals to improve their communication skills. The second article “*Practical measures for dealing with the struggles of nurses caring for people with amyotrophic lateral sclerosis (ALS) comorbid with cognitive impairment in Japan*,” by Ushikubo et al., is aimed to assess the practical measures that nurses had already implemented or wanted to propose regarding care delivery for the ALS targeted. Results underline that guideline and care manual establishment may lead to improved care delivery and to the unification of care deliveries to respond to patients' strong persistency. The third article “*Spirituality during the COVID-19 pandemic: An online creative arts intervention with photocollages for older adults in Italy and Israel*” by Keisari et al. examines how expressions of spirituality were stimulated and reflected in an online creative arts intervention for older adults during COVID-19 lockdowns. The findings illustrate how creative arts intervention guided by the tenets of dignity therapy can contribute to the spiritual care of older adults during periods of social isolation, or to the spiritual support provided in palliative care. The fourth article “*The intervention areas of the psychologist in pediatric palliative care: a retrospective analysis*” by Santini et al. is a retrospective monocentric project consisting of an analysis of characteristics of psychological interventions in a pediatric palliative care (PPC) service. The analysis show how the intervention of the psychologist in PPC does not concern only end-of-life, but a series of topics that are significant for the family to guarantee psycho-social well-being oriented toward the best quality of life. The fifth article “*Developing a Model for the Establishment of Hospice Care Delivery System for Iranian the adult patients with cancer*” by Beiranvand et al. develops a model for establishing hospice care delivery system for the adult patients with cancer. Results describe seven major domains, including the need to provide a variety of settings and services, comprehensive care plan, integration into the health system, specialized manpower, organizing the accountability system, laying the groundwork in the health system, and capacity building in the community. The sixth article “*Explaining caregivers' perceptions of palliative care unmet needs in Iranian Alzheimer's patients: A qualitative study*” by Ashrafizadeh et al. describes the perception of formal and informal caregivers of the unmet needs of Iranian Alzheimer's patients. The findings provide a deep understanding of the unmet needs of Alzheimer's patients in Iran.

## Author contributions

All authors listed have made a substantial, direct, and intellectual contribution to the work and approved it for publication.

## Conflict of interest

The authors declare that the research was conducted in the absence of any commercial or financial relationships that could be construed as a potential conflict of interest.

## Publisher's note

All claims expressed in this article are solely those of the authors and do not necessarily represent those of their affiliated organizations, or those of the publisher, the editors and the reviewers. Any product that may be evaluated in this article, or claim that may be made by its manufacturer, is not guaranteed or endorsed by the publisher.
